# Metabolite Characterization and Correlations with Antioxidant and Wound Healing Properties of Oil Palm (*Elaeis guineensis* Jacq.) Leaflets via ^1^H-NMR-Based Metabolomics Approach

**DOI:** 10.3390/molecules25235636

**Published:** 2020-11-30

**Authors:** Mohamad Shazeli Che Zain, Soo Yee Lee, Nadiah Mad Nasir, Sharida Fakurazi, Khozirah Shaari

**Affiliations:** 1 Natural Medicines and Products Research Laboratory (NaturMeds), Institute of Bioscience, Universiti Putra Malaysia, Serdang 43400, Malaysia; shazelizain@gmail.com (M.S.C.Z.); daphne.leesooyee@gmail.com (S.Y.L.); 2 Department of Chemistry, Faculty of Science, Universiti Putra Malaysia, Serdang 43400, Malaysia; nadiahmadnasir@upm.edu.my; 3 Laboratory of Vaccines and Biomolecules (VacBio), Institute of Bioscience, Universiti Putra Malaysia, Serdang 43400, Malaysia; sharida@upm.edu.my

**Keywords:** *Elaeis guineensis* Jacq., oil palm leaf, metabolite profile, solvent extract, antioxidant, wound healing, ^1^H-NMR metabolomics

## Abstract

Oil palm (*Elaeis guineensis* Jacq.) leaflets (OPLs) are one of the major agricultural by-products generated from the massive cultivation of Malaysian palm oil. This biomass is also reported to be of potential value based on its health-improving effects. By employing proton nuclear magnetic resonance (^1^H-NMR) spectroscopy combined with multivariate data analysis (MVDA), the metabolite profile of OPLs was characterized and correlated with their antioxidant and wound healing properties. Principal component analysis (PCA) classified four varieties of extracts, prepared using solvents ranging from polar to medium polarity, into three distinct clusters. Cumulatively, six flavonoids, eight organic acids, four carbohydrates, and an amine were identified from the solvent extracts. The more polar extracts, such as, the ethyl acetate-methanol, absolute methanol, and methanol-water, were richer in phytochemicals. Based on partial least square (PLS) analysis, the constituents in these extracts, such as (+)-catechin, (−)-epicatechin, orientin, isoorientin, vitexin, and isovitexin, were strongly correlated with the measured antioxidant activities, comprising ferric reducing antioxidant power (FRAP), 2,2-diphenyl-1-picrylhydrazyl (DPPH), and nitric oxide (NO) free radical scavenging activities, as well as with cell proliferation and migration activities. This study has provided crucial evidence on the importance of these natural antioxidant compounds on the wound healing properties of OPL.

## 1. Introduction

Oil palm (*Elaeis guineensis* Jacq.) leaflets (OPLs) are considered as an underutilized agricultural waste, though approximately 21.7 million tonnes of OPLs are produced annually from the Malaysian oil palm industry [1]. Studies have revealed the potential benefits of this biomass, and have eventually led to the use of OPLs as adsorbents [2], composites [3], soil fertility enhancers [4], fuels [5], papermaking materials [6], and ruminant feedstock [7]. Recently, studies have also reported OPL to contain phenolic compounds, and hence an alternative source of bioactive constituents for nutraceutical and pharmaceutical industries [8,9].

Solvent selection is one of the crucial contributors for effective extraction of phytoconstituents from plants [10]. It is necessary to consider the solvent’s polarity, reactivity, and ease of removal after extraction [11], as these factors contribute directly to the types of compounds that can be extracted, the extractable amount, and, hence, the biological activity of the extracts [12,13]. Therefore, before selecting the most suitable solvent for extracting target compounds of interest, it is necessary to compare the compositions and the bioactivity of the extractable constituents obtained from the different solvents [14,15].

Metabolomics is a robust technique for the study of small-molecule metabolite profiles of a plant matrix [16]. It is one of the fastest developing research areas, utilizing mostly high-field proton nuclear magnetic resonance spectroscopy (^1^H-NMR) or liquid chromatography mass spectrometry (LC-MS) as analytical platforms. With the use of multivariate statistical analysis (MVDA), samples can be differentiated based on their metabolite compositions. Through the interpretation of spectral data, and further comparison with databases and literature data, the important differentiating metabolites can be identified [17]. Thus, this technique is a highly promising tool for quality control and standardization of natural products [18]. The results obtained are highly reproducible and, more interestingly, MVDA in metabolomics studies also allows the identification of potential bioactive metabolites through correlations between the detected metabolites with the specific biological activity [19]. The utilization of ^1^H-NMR-based metabolomics for untargeted analysis to evaluate the correlation of known metabolites in plant extracts to their biological activities have been reported by several studies in the past such as on *Lysilomo latisiliquum* [20], *Neptunia oleracea* [21], and *Aquilaria malaccensis* Lamk [22].

Some aspects of metabolomics analysis of OPL have been previously reported by Vargas et al., [23] where they employed ultra-high-performance liquid chromatography-tandem mass spectrometry (UHPLC-MS/MS), as the analytical platform, to understand how transportation/storage conditions and extraction solvent affected its metabolite profiles [23]. However, to the best of our knowledge, there have been no studies conducted investigating the correlations between the multitude of metabolites in the OPL extract with any biological activity. In the present work, using ^1^H-NMR-based metabolomics approach, we performed a discriminative analysis of OPL extracted with solvents of different polarity (absolute methanol, aqueous methanol, ethyl acetate-methanol and ethyl acetate), identified the differentiating metabolites, and further examined the correlations between the OPL metabolites to their antioxidant and wound healing activities. The predicted bioactive metabolites were also relatively quantified.

## 2. Results

### 2.1. Visual Inspection of ^1^H-NMR Spectra of OPL Extracts and Metabolite Identification

**Figure 1:**
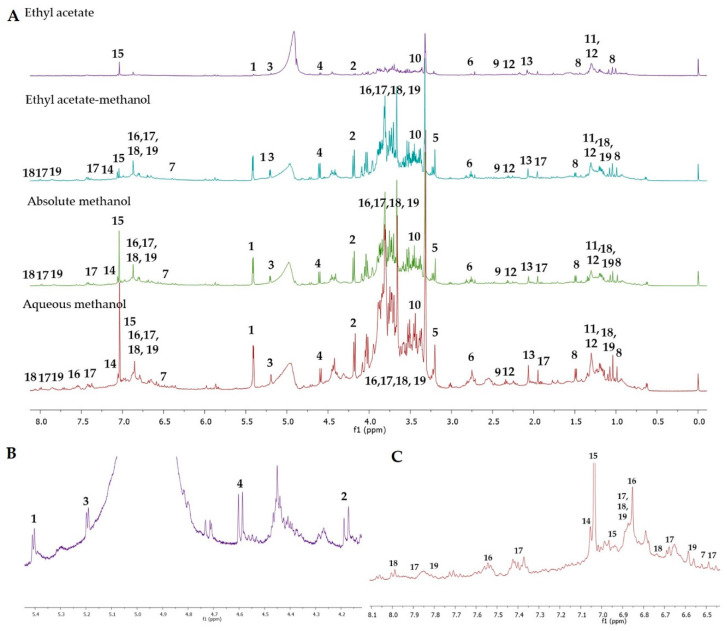
(**A**) Representative proton nuclear magnetic resonance (^1^H-NMR) spectra (500 MHz, methanol-*d*_4_) of different solvent extracts of oil palm leaflet (OPL). (**B**) Part of ^1^H-NMR spectra of ethyl acetate extract within range of 4.2 to 5.4 ppm. (**C**) Part of ^1^H-NMR spectra of aqueous methanolic extract within range of 6.5 to 8.1 ppm. Identified metabolites: **1**, sucrose; **2**, fructose; **3**, α-glucose; **4**, β-glucose; **5**, choline; **6**, citric acid; **7**, fumaric acid; **8**, palmitic acid; **9**, succinic acid; **10**, aconitic acid; **11**, fatty acid; **12**, α-linolenic acid; **13**, acetic acid; **14**, (−)-epicatechin; **15**, (+)-catechin; **16**, orientin; **17**, isoorientin; **18**, vitexin; **19**, isovitexin.

The representative ^1^H-NMR spectra of OPL samples extracted by different polarities of solvents is displayed in Figure 1. Previous studies on OPL have reported primary and secondary metabolites, which are organic acids, amino acids, simple carbohydrates, phenolic acids, luteolin, and apigenin derivatives [8,9,23,24,25,26]. Nevertheless, the data with regard to the secondary metabolites present in OPL are still considered limited. The peak assignment for signals in the 1D spectra were achieved based on comparisons with literature data [21,27,28,29,30,31,32,33,34,35,36,37,38,39,40,41,42,43,44,45] and freely available online databases, including the Nuclear Magnetic Resonance Database (NMRDB) [46], Human Metabolome Database (HMDB) [47], and PubChem [48]. Basically, 3 regions of chemical importance were visually analyzed in the ^1^H-NMR spectra of OPL extracts prepared at different extraction solvent, viz., high-field (0.9–3.50 ppm), middle-field (3.50–5.45 ppm) and low-field regions (6.0–8.2 ppm). As expected, overlapping peaks make peak assignment of the compounds very challenging. Nevertheless, signals for 19 metabolites could be discerned from careful visual inspection of the spectra. Additionally, 2D experiments (*J*-resolved) provided additional information on signal splitting (Figure S1 in Supplementary Materials). The ^1^H-NMR spectra revealed the presence of metabolites from different classes including amino acids, organic acids, carbohydrates, and flavonoids. Collectively, 6 flavonoids, 8 organic acids, 1 amino acid, and 4 carbohydrates were recognized. The characteristic ^1^H-NMR chemical shifts corresponding to these recognized metabolites are presented in Table 1.

On the whole, the efficiency of the solvents when extracting OPL metabolites improved with increasing polarity. The highly polar solvents were able to extract most of the carbohydrate metabolites, which could be assigned from the signals observed in the chemical shift region of 3.0–5.5 ppm [53]. The chemical shifts 5.42, 4.18, 5.18 and 4.62 ppm were recognized as sucrose, fructose, α-glucose, and β-glucose, respectively. The presence of these carbohydrates were in agreement with previous studies on oil palm leaf chemical composition [25,54]. The signals for these carbohydrates were also discernable in ethyl acetate, a medium polar solvent, albeit in lower quantities. The medium and highly polar solvents were also able to extract organic acids and the nutrient, choline. Except for fumaric acid, which was identified from the characteristic olefinic proton singlet at 6.54 ppm, the chemical shifts of the organic acids (citric acid, palmitic acid, succinic acid, aconitic acid, fatty acid, α-linolenic acid, and acetic acid) were identified based on their signals observed in the aliphatic region (0.90–3.50 ppm). These organic acids were detected in all the solvent extracts, except fumaric acid which was absent in ethyl acetate extracts.

**Figure 2:**
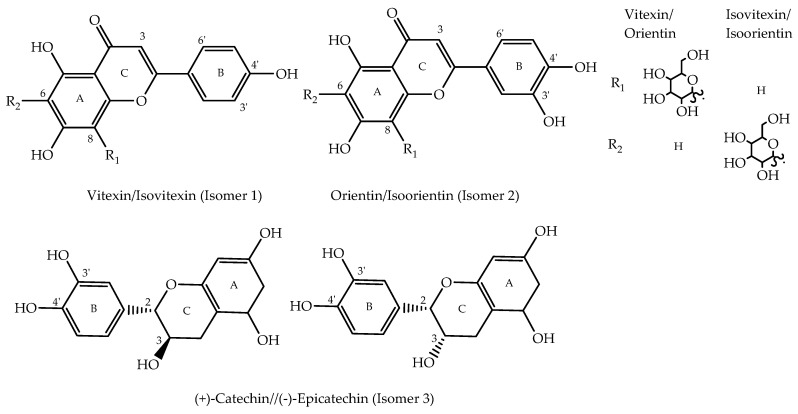
Major flavonoids detected in OPL extracts. Isomer 1, vitexin (**18**)/isovitexin (**19**); Isomer 2, orientin (**16**)/isoorientin (**17**), and Isomer 3, (+)-catechin (**15**)/(−)-epicatechin (**14**).

Extracts prepared from methanol, either on its own (absolute) or as admixtures with ethyl acetate or water, showed proton signals characteristic of flavonoids, specifically those in the downfield region between 6.00 and 8.00 ppm. Among the major flavonoids detected in these extracts were apigenin, luteolin, and catechin derivatives, in accordance with our previous findings using UHPLC-MS/MS analysis [25,26]. The flavonoids were detected as isomeric pairs—i.e., vitexin with isovitexin, orientin with isoorientin, and catechin with epicatechin (Figure 2). The chemical shifts 8.02, 6.89, and 6.77 ppm were assigned to vitexin while signals at 7.79, 6.88, and 6.52 ppm were assigned to isovitexin. The signals resonating at 7.54, 6.86, and 6.63 ppm were assigned to orientin while signals detected at 7.40, 6.89, 6.67, and 6.48 ppm were assigned to isoorientin. The proton signals of orientin and isoorientin were lower than those exhibited by the protons of vitexin and isovitexin at the same positions. Compared to vitexin and isovitexin, orientin and isoorientin experienced a shielding effect due to the additional hydroxyl group on ring B (Figure 2). This additional substituent acted as an electron donating group which increased the electron density of the nucleus, resulting in a slight shielding effect and hence lower chemical shifts. Intense peaks between 3.00 to 4.00 ppm were observed which were assigned to the sugar moiety attached to the aglycone of identified flavonoids. The peak assignments were based on comparison with literature data (Table 1) and confirmed with chemical shift data of commercial standards (Figure S2).

Meanwhile, the catechin/epicatechin isomeric pair was detected based on the chemical shifts occurring at 7.04, 7.01, and 6.95 ppm. The peaks at 7.04 and 6.95 ppm were assigned to (−)-epicatechin, while the peak at 7.01 ppm was assigned to (+)-catechin. These signals assignment were in agreement with the chemical shifts reported previously [50]. The difference between (+)-catechin and (−)-epicatechin is the different configurations of C-2 and C-3. (+)-Catechin is the isomer with a *trans*-configuration while (−)-epicatechin has a *cis*-configuration (Figure 2). The *trans*-related H-2 and H-3 protons of epicatechin were slightly more deshielded in comparison to the *cis*-related protons of catechin.

### 2.2. Classification of OPL Extracts by Principal Component Analysis (PCA)

Variation in metabolites in the different solvent extracts was examined by multivariate analysis (MVDA). The ^1^H-NMR datasets of the aqueous methanol, absolute methanol, ethyl acetate-methanol and ethyl acetate extracts were subjected to PCA to primarily distinguish between the extracts, and to identify the discriminating factors. The PCA model was obtained with good fitness (R^2^X) and high predictability (Q^2^) values of 0.995 and 0.960, respectively. There were no notable outliers. The first principal component (PC1) provided 79.7% of the variance, while the second principal component (PC2), gave 7.23%, cumulatively describing a total variance of 86.93%.

**Figure 3:**
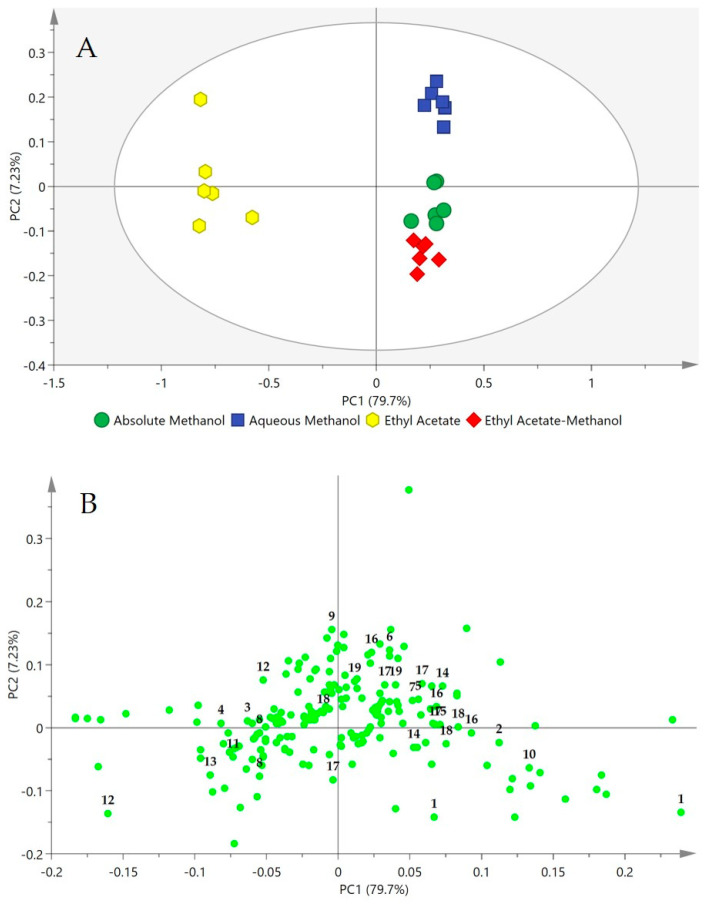
Principal component analysis (PCA) score (**A**) and loading (**B**) plots constructed from ^1^H-NMR spectral data of four different solvent extracts of OPL. The plot ellipse represents 95% hoteling T2 confidence. Identified metabolites: **1**, sucrose; **2**, fructose; **3**, α-glucose; **4**, β-glucose; **5**, choline; **6**, citric acid; **7**, fumaric acid; **8**, palmitic acid; **9**, succinic acid; **10**, aconitic acid; **11**, fatty acid; **12**, α-linolenic acid; **13**, acetic acid; **14**, (−)-epicatechin; **15**, (+)-catechin; **16**, orientin; **17**, isoorientin; **18**, vitexin; **19**, isovitexin.

The PCA score plot (Figure 3A) showed that different solvent extracts were grouped into three clusters with no notable outliers. The ethyl acetate extract was well separated from the methanolic extracts (aqueous methanol, absolute methanol, and ethyl acetate-methanol) by PC1. The absolute methanol and ethyl acetate-methanol extracts were also projected closer to each other, rather than with the aqueous methanol extract. This indicated that the phytochemical constituents in the absolute methanol and ethyl acetate-methanol extracts were very similar. The same metabolites may have been extracted by the two solvent systems but in varying quantities.

**Figure 4:**
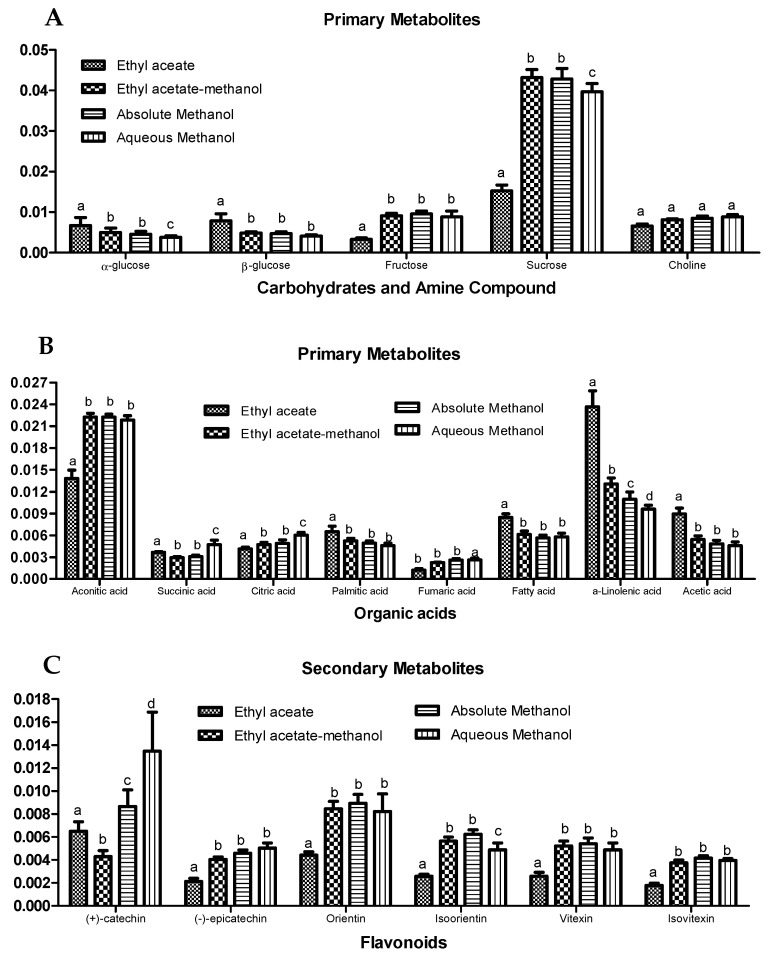
Relative quantification of identified compounds in different solvent extracts of OPL based on the mean peak areas of selected ^1^H-NMR signals. (**A**) Carbohydrates and amines; (**B**) organic acids; (**C**) flavonoids. The discriminating chemical shifts (ppm) employed for the relative quantification were sucrose (δ 3.82), fructose (δ 4.18), α-glucose (δ 5.18), β-glucose (δ 4.62), choline (δ 3.20), citric acid (δ 2.74), succinic acid (δ 2.54), aconitic acid (δ 3.42), α-linolenic acid (δ 1.30), acetic acid (δ 2.07), palmitic acid (δ 0.9), fatty acid (δ 1.34), (+)-catechin (δ 7.02), (−)-epicatechin (δ 6.94), orientin (δ 7.54), isoorientin (δ 7.40), vitexin (δ 8.02), and isovitexin (δ 7.79). Bars labelled with the different letters (a,b,c,d) are significantly different between solvent extracts for the same metabolites (*p* < 0.05).

The discriminating metabolites between the various clusters were determined from the loading plot (Figure 3B). The ethyl acetate extract contained higher amounts of α- and β-glucose, acetic acid, and fatty acids, notably, the essential fatty acid α-linolenic acid. Meanwhile, the methanolic solvent systems were able to extract a large number of compounds, comprising moderately polar to polar molecules. The methanolic solvent systems extracted both primary (viz. succinic acid, citric acid, aconitic acid, fumaric acid, choline, sucrose, and fructose) and secondary metabolites (viz. apigenin, luteolin, and catechin derivatives). However, although the ethyl acetate-methanol and absolute methanol extracts appeared to be in a separate cluster from the aqueous methanol extract in the score plot, there were no obvious discriminants between these three solvent extracts. The loading plot showed a congregation of most of the ^1^H-NMR chemical shifts close to PC2. Overall, as expected, the PCA results revealed that the methanolic solvent systems were capable of liberating both primary and secondary metabolites from OPL, while ethyl acetate mainly extracted some primary metabolites. Relative quantification of the identified metabolites (Figure 4) showed no significant difference in their extractability when methanolic solvent systems were used, while ethyl acetate, representing a less polar system, was mainly efficient for extraction of the fatty acids, particularly α-linolenic acid. The results indicated that the polar solvent systems were the better solvents for extraction or recovery of most OPL metabolites, especially the flavans and the four major OPL flavonoids.

### 2.3. Influence of OPL Extracts on Polyphenolic Contents, Antioxidant, and Wound Healing Properties

As shown in Table 2, the phenolic contents, antioxidant and wound healing properties are influenced by the choice of solvent used for extraction. For total phenolic content (TPC), there was a positive relationship with solvent polarity, where the TPC increased with increasing solvent polarity. Aqueous methanol extract had the highest TPC, with a value of 393.61 mg GAE/g extract, followed by absolute methanol and ethyl acetate-methanol extracts, respectively. On the other hand, the absolute methanol extract showed the highest Total Flavonoid Content (TFC) with a value of 135.40 mg QCE/g extract, followed by aqueous methanol, and ethyl acetate-methanol extracts. Meanwhile, the ethyl acetate extract had the lowest TPC and TFC values, with 121.71 mg GAE/g and 5.94 mg QCE/g extract, respectively. Statistically, there were no significant differences (*p* < 0.05) in the TFC extracted by the three methanolic solvents. These results suggested that the methanolic solvent systems are more efficient for the extraction of polyphenols in OPL.

The results of the antioxidant assays on the solvent extracts were similar to the trend of the polyphenolic contents, where the methanolic extracts exhibited significantly greater activities in comparison with ethyl acetate extracts. The methanolic extracts exhibited high ferric antioxidant power (FRAP) with values between 71.62 and 101.48 mg AAE/g extract. There was also no significant difference between the values. For the 2,2-diphenyl-1-picrylhydrazyl (DPPH) and nitric oxide (NO) free radical scavenging activities, the low IC_50_ values revealed the potency of the methanolic extracts. The aqueous and absolute methanol extracts showed the most significant scavenging of DPPH free radicals with IC_50_ values of 3.53 and 6.54 µg/mL, respectively. Meanwhile, the aqueous methanol extract recorded the lowest IC_50_ value of 18.77 µg/mL in the NO free radical scavenging, followed by absolute methanol with IC_50_ values of 67.64 µg/mL. These findings emphasized the efficiency of methanol for the extraction and recovery of antioxidant principles from OPL and are in agreement with Tahir et al. [8].

The various solvent extracts of OPL were also evaluated for their wound healing properties by assessing their efficacy in enhancing the proliferation and migration of 3T3 cells. Prior to this, to find the optimum concentration of extracts for cell proliferation and migration, various concentrations, ranging from 1.56 to 100 µg/mL, were preliminarily screened. The cells proliferated and migrated rapidly at a low concentration of 1.56 µg/mL. The results of cell proliferation and migration activities at this extract concentration are presented in Table 2. With the high cell proliferation activity (>95%), the results indicated that all the OPL extracts did not affect the cell viability of 3T3 cells, besides promoting cell growth. Meanwhile, a wound scratch test assay was conducted to assess the migration of cells. The results revealed that methanolic extracts showed high cell migration activity, enhancing the 3T3 cells’ migration at percentages ranging from 85.83 to 93.34%, with no significant difference between them. Overall, methanolic extracts were found to possess good cell proliferation and migration activities.

Pearson’s correlation was performed to assess the relationship between the polyphenolic contents and the bioactivities. The results are presented in Table 3. There were positive correlations between the phenolic contents and the bioactivities, with correlations between TPC and cell proliferation, between TFC and DPPH free radical scavenging activity, and between TFC and cell migrations as the most significant ones. This strong positive relationship revealed the contribution of phenolic constituents, particularly the flavonoids, to their respective bioactivities. The promotion of cell proliferation by phenolic compounds and DPPH scavenging and cell migration enhancement by flavonoids have been reported previously [26,55,56]. In addition, the Pearson’s correlation also revealed a strong correlation between antioxidant and wound healing activities. These results could provide information on the role of free radical scavenging as one of the important events in wound healing [57]. Although Pearson’s correlation showed that the phenolic constituents contributed to the antioxidant and wound healing activities of the OPL extracts, the individual metabolites that are responsible for the bioactivities require further investigation.

### 2.4. Correlation between Identified Metabolites and Antioxidant and Wound Healing Activities of OPL Extracts

To comprehend the relationship between the individual metabolites and the bioactivities, the correlation was further extended by computing the supervised MVDA. Partial least squares (PLSs) projection to latent structures was applied to determine the chemical constituents that are correlated to the bioactivities. PLS can relate the data of independent variables (X-matrix, ^1^H-NMR spectral data) to the data of dependent variables (antioxidant and wound healing activities), hence the potential biomarkers can be predicted. The PLS model obtained from the NMR spectral and bioactivity data of OPL extracts was validated by means of internal cross validation, permutation test, and regression analysis. Autofit of the PLS model in SIMCA revealed a good fitness (R^2^Y = 0.868) and predictive ability (Q^2^ = 0.60) of the model (Figure S3). The permutation plots presented in Figure S4 revealed that there was no overfitting of the PLS model. The Y-intercepts of R^2^ and Q^2^ were less than 0.3 and 0.05, respectively. These data indicated that the PLS model showed a satisfactory level of validity [41,42].

**Figure 5:**
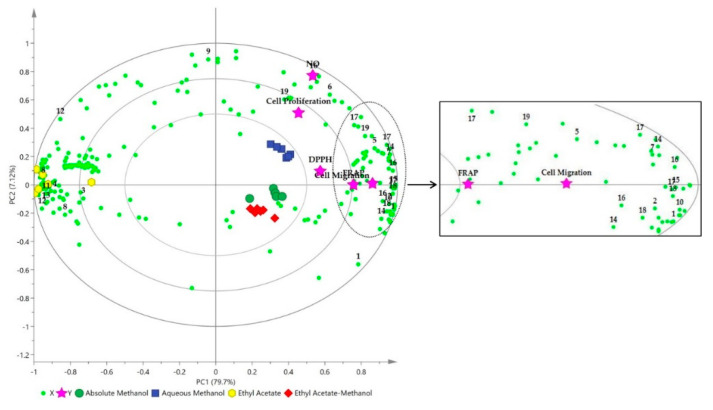
Partial least square (PLS) loading biplot of bioactivities represented by ferric reducing antioxidant power (FRAP), 2,2-diphenyl-1-picrylhydrazyl (DPPH), and nitric oxide (NO) free radical scavenging activities, cell proliferation, and cell migration. Identified metabolites: **1**, sucrose; **2**, fructose; **3**, α-glucose; **4**, β-glucose; **5**, choline; **6**, citric acid; **7**, fumaric acid; **8**, palmitic acid; **9**, succinic acid; **10**, aconitic acid; **11**, fatty acid; **12**, α-linolenic acid; **13**, acetic acid; **14**, (−)-epicatechin; **15**, (+)-catechin; **16**, orientin; **17**, isoorientin; **18**, vitexin; **19**, isovitexin.

Similar to the PCA score plot, in the PLS biplot, the OPL extracts were separated into three clusters, as shown in Figure 5. The methanolic extracts were clustered on the positive side of PC1 while the ethyl acetate extract was on the negative side. All the Y variables (DPPH, NO, FRAP, cell proliferation, and cell migration activities) were projected on the same side as the methanolic extracts, demonstrating their better activities compared to the ethyl acetate extract. These results were consistent with the bioactivitiy results discussed previously. The phenolic constituents, particularly the flavans and flavonoids namely (+)-catechin, (−)-epicatechin, orientin, isoorientin, vitexin, and isovitexin, were located close to the Y variables, revealing their significant contribution towards the antioxidant and wound healing activities of OPL. These results supported the Pearson’s correlation results. Previous studies have reported that different plant matrices containing catechin, apigenin, and luteolin derivatives exhibited arrays of bioactivities comprising antioxidant, anti-inflammatory, and wound healing activities [8,25,26,55,58]. The primary metabolites comprising carbohydrates (sucrose and fructose) and organic acids (citric acid, succinic acid, fumaric acid, and aconitic acid) were also shown to be correlated with the antioxidant and wound healing activities. These findings are in accordance with other studies that reported primary metabolites, such as organic acids and carbohydrates, also contribute to antioxidant and wound healing activities of a plant extract [59,60].

## 3. Discussion

Overall, the findings revealed the methanolic extracts were rich in secondary metabolites, namely flavonoids, as displayed in proton NMR spectra. Both Pearson and PLS correlation studies demonstrated these groups of compounds were strongly correlated with studied antioxidant and wound healing properties. The role of these flavonoids as natural antioxidants often relates to their potential scavenging activity in combating excessive oxidative stress during wound healing. In normal metabolism, the level of oxidants and antioxidants is maintained; however, overproduction of oxidants can lead to oxidative damage that may spread all over the targeted cells such as DNA, lipids, and proteins. Hence, the presence of antioxidant molecules is able to shield these targeted cells at the risk of damage by free radicals or known as reactive oxygen species (ROS) such as super anion radical (O2•), singlet oxygen (O•), nitric oxide (NO•), hydroxyl radical (HO•), peroxynitrite (ONOO•), and hydrogen peroxide (H_2_O_2_) [61]. Studies reported that wounds cause the overproduction of ROS during wound repair processes as a defence mechanism against invading bacteria resulting in severe tissue damage at the targeted cells and even lead to neoplastic transformation which reducing the wound healing process [62,63]. Conversely, the antioxidant defence system works efficiently for neutralizing these free radicals by undergoing three main phases. Firstly, metal chelating proteins and enzymatic antioxidants including peroxidases will stop the production of free radicals followed by scavenging the free radicals by non-enzymatic or radical scavenging antioxidants to ensure no formation of oxidation chain. Lastly, enzymes such as lipases, proteases, and DNA repair enzymes will have roles in repairing and replacing the damages of the targeted cells and to reconstitute the damaged membranes [61].

**Figure 6:**
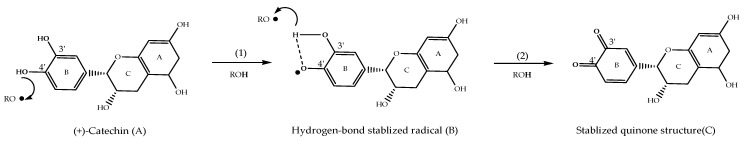
Schematic diagram of hydrogen transfer mechanism in (+)-catechin. (**A**): (+)-Catechin; (**B**): Hydrogen bond stabilized radical; (**C**): Stabilized quinone structure.

The antioxidant activities expressed by flavonoids could be related to their structure-antioxidant activity relationships. The presence of one or more phenolic hydroxyl groups (-OH) in their structure plays a significant role in these activities [59,64,65,66]. The contribution of flavonoids, especially catechin, luteolin, and apigenin derivatives, in antioxidant action can be explained by direct scavenging of ROS via hydrogen atom donation, which is one of the common antioxidant action mechanisms of flavonoids [67]. To participate in the mechanism, the flavonoid structure should have an *ortho*-dihydroxy substitution in the B ring and C2-C3 double bond, or C4 carbonyl group in the C ring [67]. Flavanols (catechin isomers) and flavones (vitexin and orientin isomers) contain hydroxyl groups attached to carbons C4′ and/or C3′ of ring B (Figure 2). The hydroxyl groups attached to these positions could actively participate in the hydrogen transfer mechanism. For example, in the case of (+)-catechin (A), the hydrogen atom of the C4′-OH group was donated to the radical moiety (RO•) (Figure 6). The resulting unstable oxygen atom on C4′ then formed a hydrogen bond with hydroxyl group on C3′ forming a stabilized catechin radical intermediate (B). Then, the catechin radical stabilized a second radical moiety (RO•) by donating the hydrogen atom of the C3′-OH, forming the stable quinone structure C.

## 4. Materials and Methods

### 4.1. Reagents, Solvents, and Commercial Standards

Analytical grade absolute methanol and ethyl acetate, deuterated methanol-*d*_4_ (CH_3_OH-*d*_4_), non-deuterated KH_2_PO_4_, sodium deuterium oxide (NaOD), trimethylsilyl propionic acid-*d*_4_ sodium salt (TSP) and deuterium oxide (D_2_O) were supplied by Merck (Darmstadt, Germany). Water was purified by a Milli-Q system (Millipore Lab, Bedford, MA, USA). The polyphenolic contents and antioxidant assays reagents (DPPH powder, Folin-Ciocalteu, sodium acetate, 2,4,6-tris(2-pyridyl)-*S*-triazine (TPTZ), ferric chloride, and sodium carbonate) and commercial standards (quercetin, gallic acid, and ascorbic acid) were purchased from Sigma (Aldrich, Darmstadt, Germany), while aluminum chloride was provided by HmbG Chemicals (Hamburg, Germany). Conventional organic solvents such as ethanol, methanol, ethyl acetate, and dimethyl sulfoxide (DMSO) were supplied by R&M Chemicals (Essex, UK). Reagents for wound healing assays such as trypsin and penicillin-streptomycin were bought from Nacalai Tesque (Kyoto, Japan), whereas phosphate buffer saline (PBS) and Dulbecco’s modified eagle medium (DMEM) were obtained from Solarbio (Beijing, China). Fetal bovine serum (FBS) and cell counting kit (CCK-8) were supplied by Tico Europe (Amstelveen, Netherlands) and Dojindo Lab (Kumamoto, Japan), respectively. The commercial standards of vitexin, orientin, isovitexin, and isoorientin with a purity of more than 98% were provided by ChemFaces (Wuhan, China).

### 4.2. Sampling and Preparation of Extracts

**Figure 7:**
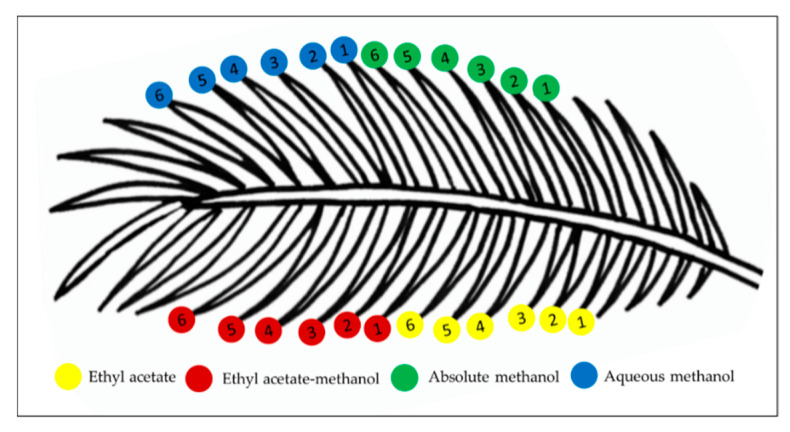
Illustration of sampling method of the 24 mature leaflets from 17th oil palm frond of *Elaeis guineensis* Jacq. Six leaflets were sampled for each solvent system used for extraction.

The leaflets of *Elaeis guineesis* Jacq. were harvested from the University Agricultural Park, UPM, Malaysia. A piece of leaflet was sent for authentication to the Institute of Bioscience, UPM with the specimen number SK 3332/1. To ensure uniformity in samples, the sampling method routinely practiced by agronomists was followed [68]. The leaflets were obtained from the 17th frond from the apex of the tree. To minimize variation, the leaflets were harvested in the morning (8.00 am). A total of 24 uniform-sized leaflets were detached from the petiole of the oil palm frond and labelled accordingly, as shown in Figure 7. The leaflets were cleaned with water, their rachises were removed, and then were cut into small pieces. The leaflets were then oven-dried at 35 °C, until a constant weight was reached. The dried leaflets were ground and sieved to a fine powder with uniform size.

The OPL powdered samples were extracted with four different solvent systems—i.e., ethyl acetate (100%), ethyl acetate-methanol (1:1), absolute methanol (100%), and methanol-water (4:1). For each of the solvent system, 0.1 g of OPL powder, placed in a 50 mL centrifuge tube, was mixed with 5 mL solvent, and vortexed for 0.5 min at the speed of at 3000 rpm. The extraction was performed using an ultrasonication method for 30 min at 25 °C in ultrasonic bath with frequency of 40 Hz. To obtain the crude dried extract, the mixture was centrifuged (4000× rpm, 15 min), and the supernatant was concentrated using vacuum evaporation and freeze-dried (Kansas City, MO, USA). A total of six replicates for each solvent system was prepared.

### 4.3. NMR Sample Preparation, Measurement, and Spectra Processing

The sample preparation and NMR measurements were executed according to established protocol [40] with some modifications. For each sample, a 20 mg weight of the extract was placed into an Eppendorf tube, and mixed with an equal volume (300 µL) of deuterated methanol and prepared buffer (pH 6.0 and 0.01% TSP). Prior to analysis, the mixture was homogenized by an ultrasonication technique (15 min, 25 °C) and subsequently centrifuged for 15 min at a speed of 4000 rpm. For ^1^H-NMR spectral acquisition, 550 µL of filtered extract solution was pipetted into a clean NMR tube.

The spectra were retrieved from a Varian INOVA NMR spectrometer (spectrometer (Varian Inc., Palo Alto, CA, USA)) with a frequency of 500 MHz. For each 1D spectrum, the conditions of the instrument were set at 24 °C with 64 scans, a 3.54 min acquisition time and 2 s relaxation delay. To suppress the water signal, a pre-saturation (PRESAT) pulse sequence was employed. The conditions produced a spectral width of −2 to 14 ppm.

The ^1^H-NMR spectra of all extracts were processed using Chenomx software (Edmonton, AB, Canada). The spectral region (0.50–10.00 ppm) was bucketed into 243 integrated regions with δ 0.04 bin size. The signals of residual water (δ 4.70–4.88 ppm) and methanol (δ 3.24–3.33 ppm) were excluded during binning. To assist peak assignment, the 2D NMR experiment (*J*-resolved) was performed. For further confirmation, the assigned signals of orientin, vitexin, isoorientin, and isovitexin were verified with commercial standards (Figure S2). The data processing was carried out using MestRenova (Mestrelab Research, Santiago de Compostella, Spain).

The identified primary and secondary metabolites were relatively quantified by plotting their respective mean peak area of the assigned signals observed in the ^1^H-NMR spectra. The discriminatory metabolites were selected based on the NMR signals which were not overlapping with other signals for the relative quantification, performed using SPSS software (SPSS Inc., Chicago, IL, USA).

### 4.4. Total Phenolic (TPC) and Flavonoid Content (TFC) Determination

The TPC assay was executed using Folin-Ciocalteu (F-C) approach [69], with slight adjustments. Briefly, 0.1 mg/mL extract solution was prepared and an aliquot of 20 µL was mixed with 100 µL F-C reagent and 80 µL sodium carbonate solution (7.5%) in each well of a 96-well microtiter plate. The reaction was allowed to happen within 30 min incubation prior to the absorbance measurement at 750 nm using a microplate reader (Tecan Group Ltd., Männedorf, Switzerland). The TPC assay was carried out in six replicates for each extract and polyphenolic contents of the samples were evaluated from a calibration curve constructed for a series of gallic acid concentrations. The TPC values were expressed in milligrams of gallic acid equivalents per gram of extract (mg GAE/g extract).

The TFC assay was executed using an aluminum chloride complex forming method [25]. Briefly, 0.1 mg/mL of extract solution was prepared and an aliquot of 125 µL was mixed with 375 µL 95% ethanol, 25 µL aluminum chloride solution (10%), 25 µL sodium acetate solution (1M), and 700 µL water in a 2 mL Eppendorf tube. Following this, 200 µL of the mixture was transferred to 96-well microtiter plate and incubated for 40 min at 25 °C. The absorbance measurement was performed using a microplate reader at 415 nm. The TFC assay was performed in six replicates for each extract and the flavonoid contents were calculated from a calibration curve constructed for a series of quercetin concentrations. The TFC values were stated in milligrams of quercetin equivalents per gram of extract (mg QCE/g extract).

### 4.5. In Vitro Antioxidant Assays

The antioxidant activity of the solvent extracts were performed by ferric reducing antioxidant power (FRAP), 2,2-diphenyl-1-picrylhydrazyl (DPPH), and nitric oxide (NO) free radical scavenging assays using the following reagents: 2,4,6-tris(2-pyridyl)-1,3,5-triazine (TPTZ), DPPH reagent, *N*-(1-naphthyl)ethylenediamine dihydrochloride, and sulphanilamide. Measurements of absorbances were recorded on a microplate reader. Quercetin was employed as positive control in each assay.

FRAP assay: The solvent extracts were measured for FRAP according to a previously described method [70], with slight adjustments. To prepare 25 mL FRAP reagent, 2.5 mL TPTZ solution (10 mM) and 2.5 mL ferric chloride solution (20 mM) were mixed, and the remaining volume was topped up with 30 mM acetate buffer with pH adjustment to 3.6. Prior to use, the mixture was incubated for 15 min at 37 °C. For the FRAP assay, 20 µL of extract was mixed with 180 µL of prepared reagent and the absorbance measurement was read at 593 nm after a 30 min incubation. The FRAP assay was performed in six replicates for each extract and the quantifications were calculated from a calibration curve constructed for a series of ascorbic acid concentrations. The results were stated in micrograms of ascorbic acid equivalents per milligram of extract (µg AAE/g extract).

DPPH free radical scavenging assay: The assay on the OPL extracts was executed by following our recent published method [25]. Briefly, 0.1 mg/mL extract solution was prepared and serially diluted (100 to 0.7 µg/mL) inside 96-well microtiter plate. Subsequently, 100 µL of 0.059 mg/mL DPPH solution was added and incubated for 30 min. The absorbance measurement was read at 515 nm. The extract was carried out in six replicates and the radical inhibitions were presented as IC_50_ value in µg/mL. The scavenging activity (SA) was determined by using this formula:

SA % = [(A_o_ − A_s_)/A_o_] × 100% (1)

where A_o_ and A_s_ are absorbances of the solvent blank and extract solution, respectively.

NO free radical scavenging assay: The assay was performed using a 96-well plate format as described previously [25]. To prepare Griess reagent, 0.1 g sulphanilamide and 0.01 g *N*-(1-naphthyl)-ethylenediamine dihydrochloride were weighed and mixed with 10 mL phosphoric acid (2.5%). For the assay, 1 mg/mL of extract solution was prepared and serially diluted (1000 to 0.98 µg/mL). Subsequently, 60 µL of extract solution was mixed with 60 µL sodium nitroprusside solution and incubated for 150 min. The absorbance measurement was read at 550 nm after the addition of 60 µL prepared Griess reagent. The extract was carried out in six replicates and the radical inhibitions were presented as an IC_50_ value in µg/mL. Calculation of the SA value was carried out according to the same formula as for the DPPH assay.

### 4.6. In Vitro Wound Healing Assays

The wound healing properties of the solvent extracts were evaluated by cell proliferation and scratch (cell migration) assays, performed as described previously [26]. Allantoin was treated as the positive control for both assays. The wound healing assays were performed by using Swiss 3T3 albino mouse fibroblasts (Cell Line Service, Appelheim, Germany). A cell culture medium, comprised of high glucose DMEM, 10% FBS, and 1% penicillin-streptomycin, was used. The cells were maintained in a humidified 5% CO_2_ incubator at 37 °C. Prior to seeding, the cells at 70–80% confluence were used for all the experiments. A total of 1.563 µg/mL of solvent extracts and allantoin were prepared by mixing with a cell culture medium and filtered through 0.22 µm syringe filter for sterilization purpose.

Cell proliferation assay: The 3T3 cells were seeded in a 96-well microtiter plate at a density of 1 × 10^5^ in 100 µL culture medium per well. After 24 h incubation, the medium was replaced with 100 µL of the extract solution and incubated for a further 48 h. After this period, 10 µL of CCK-8 reagent was transferred into the mixture and continued incubating for 4 h. The absorbance of the sample, positive and negative (cells without treatment), was recorded on a microplate reader at 450 nm, against a solvent blank. Each extract solution was performed in six replicates.

Cell migration assay: The 3T3 cells were seeded in 24 well plates at a density of 3 × 10^5^ cells/mL per well. By employing a scratch assay technique, after 24 h incubation, a linear line was scratched on the confluent monolayered cells with a sterile P200 pipette tip. Care was taken when making the scratch to obtain a uniform size and length on each well. After adequately rinsing the well to eradicate the debris, the extract solution was pipetted into the well as treatment. The migration of the cell was monitored for wound closure and photographed on using phase contrast microscope, with the magnification set to 5× after 0 and 48 h of incubation. Using Image-J software (version 1.45, the area of the cells covered on the scratch area was measured and expressed as a percentage (%) of wound closure. Each extract solution was performed in six replicates.

### 4.7. Statistical Analysis

The data retrieved from polyphenolic contents (TPC and TFC), antioxidant (FRAP, DPPH, and NO scavenging activities), and wound healing properties (cell proliferation and cell migration) assays were analyzed using Minitab software (Minitab Inc, State College, PA, USA) and GraphPad Prism software (San Diego, CA, USA) where the final values were presented as mean ± standard deviation of six replicates. To determine the significant differences of each datum, a one-way analysis of variance (ANOVA) was employed. For MVDA, PCA and PLS were analyzed using SIMCA-P+ software (Umetrics AB, Umea, Sweden, version 13.0) with Pareto scaling where the proton NMR chemical shifts (metabolites) were used to represent X-variables, while the antioxidant (1/IC_50_) and wound healing results were treated as Y-variables.

## 5. Conclusions

The variation of metabolites in OPL extracts prepared using aqueous methanol, absolute methanol, ethyl acetate-methanol, and ethyl acetate as the solvent systems for extraction, as well as the correlation of the identified metabolites with the antioxidant and wound healing properties of extracts, were evaluated using a ^1^H-NMR-based metabolomics approach. A total of 19 metabolites, comprising six flavonoids, eight organic acids, four carbohydrates, and an amine, were identified in the various solvent extracts. For extraction purposes, the methanolic solvent systems (ethyl acetate-methanol, absolute methanol, and aqueous methanol) were more effective for the extraction of both primary and secondary metabolites. Based on the strong correlations with the antioxidant and wound healing activities, it could be concluded that the flavonoids (+)-catechin, (−)-epicatechin, orientin, isoorientin, vitexin, and isovitexin are the main bioactive metabolites. Several organic acids and carbohydrates constituents also contributed to the bioactivities. The findings of this study have emphasized the importance of these natural antioxidant compounds to the wound healing potential of OPL. From the perspective of quality control and assurance of therapeutic efficacy, this information will be of great value for future exploitation of this agriculture biomass into plant-based products, specifically for wound treatment purposes.

## Figures and Tables

**Table 1:** Metabolites identified in different solvent extracts of OPL and key ^1^H-NMR chemical shifts (500 MHz, methanol-*d*_4_).

No	Metabolites	^1^H-NMR Chemical Shift (Multiplicity)	Aqueous Methanol	Absolute Methanol	Ethyl Acetate-Methanol	Ethyl Acetate	Reference
1	Sucrose	5.42 (d); 3.82 (m)	+	+	+	+	[21,27,30,38,39]
2	Fructose	4.18 (d)	+	+	+	+	[21,29,39]
3	α-glucose	5.18 (d)	+	+	+	+	[21,27,30,39]
4	β-glucose	4.62 (d)	+	+	+	+	[21,29,30,39]
5	Choline	3.20 (s)	+	+	+	+	[21,31,34,39]
6	Citric acid	2.74 (d)	+	+	+	+	[29,30,39]
7	Fumaric acid	6.54 (s)	+	+	+	-	[30,31,32]
8	Palmitic acid	1.66 (m); 0.90 (t)	+	+	+	+	[27]
9	Succinic acid	2.54 (s)	+	+	+	+	[30,38]
10	Aconitic acid	3.42 (s)	+	+	+	+	[33]
11	Fatty acid	1.34 (m)	+	+	+	+	[21,39,49]
12	α-linolenic acid	1.30 (brs); 2.35 (t)	+	+	+	+	[27,29,30]
13	Acetic acid	2.07 (s)	+	+	+	+	[35,36,37]
14	(−)-epicatechin	7.04 (s); 6.95 (s)	+	+	+	+	[9,25,50,51]
15	(+)-catechin	7.01 (d)	+	+	+	-	[9,25,50,51]
16	Orientin	7.54 (s); 6.86 (s); 6.63 (s); 3.00–4.00 (m)	+	+	+	-	[8,25,26,32,39,52]
17	Isoorientin	7.40 (d); 6.89 (d); 6.67 (s); 6.48 (s); 3.00–4.00 (m); 1.96 (s)	+	+	+	-	[8,25,26,32,39,52]
18	Vitexin	8.02 (d); 6.89 (d); 6.77 (s); 3.00–4.00 (m); 1.17 (t)	+	+	+	-	[8,25,26,32,39,52]
19	Isovitexin	7.79 (d); 6.88 (d); 6.52 (s); 3.00–4.00 (m); 1.17 (t)	+	+	+	-	[8,25,26,32,39,52]

(+)—presence, (-)—absence of an identified metabolite in the OPL extract.

**Table 2:** Phenolic contents, antioxidant, and wound healing properties of different OPL extracts.

Solvent Systems	Polyphenolic Contents		Antioxidant Activities			Wound Healing Properties	
	TPC (mg GAE/g)	TFC (mg QCE/g)	FRAP (mg AAE/g)	DPPH IC_50_ (µg/mL)	NO IC_50_ (µg/mL)	Proliferation (%)	Migration (%)
Ethyl acetate	121.71 ± 32.78 ^a^	5.94 ± 3.58 ^a^	16.26 ± 6.65 ^a^	67.92 ± 14.16 ^a^	213.34 ± 58.14 ^a^	94.97 ± 2.12 ^a^	64.34 ± 1.68 ^a^
Ethyl acetate-methanol	174.19 ± 32.40 ^b^	121.48 ± 6.67 ^b^	94.00 ± 23.94 ^b^	10.74 ± 3.51 ^b^	109.08 ± 29.61 ^b^	98.41 ± 4.07 ^a^	85.83 ± 3.25 ^b^
Absolute methanol	213.08 ± 41.61 ^b^	135.40 ± 9.76 ^b^	71.62 ± 21.05 ^b^	6.54 ± 3.31 ^b,c^	67.64 ± 21.97 ^c^	100.13 ± 1.07 ^a^	88.56 ± 6.94 ^b^
Aqueous methanol	393.61 ± 36.11 ^c^	129.72 ± 8.7 ^b^	101.48 ± 16.67 ^b^	3.53 ± 1.30 ^c^	18.77 ± 3.37 ^d^	107.7 ± 13.11 ^a^	93.34 ± 4.35 ^b^

Values labelled with the different letters (a,b,c,d) are significantly different between solvent extracts in the same tested parameters (*p* < 0.05).

**Table 3:** Pearson’s coefficient of antioxidant and wound healing activities of different OPL extracts.

Activity		TPC	TFC	DPPH	NO	FRAP	Proliferation
Antioxidant	TFC	0.605					
	DPPH	0.654	0.997 *				
	NO	0.869	0.914	0.933			
	FRAP	0.705	0.921	0.945	0.887		
Wound healing	Proliferation	0.995 *	0.677	0.723	0.911	0.762	
	Migration	0.761	0.976 *	0.988 *	0.976 *	0.947	0.820

Values with asterisk (*) were significant at α = 0.05 based on Pearson’s correlation coefficient’s method.

## Supplementary Materials

The following are available online. Figure S1: 2D NMR ^1^H (*J*-resolved) spectrum of methanolic extract of oil palm (*Elaeis guineensis* Jacq.) leaflet, Figure S2: ^1^H-NMR spectra of commercial standards of orientin (**16**), isoorientin (**17**), vitexin (**18**) and isovitexin (**19**). Signals appeared in the region between 6.0–8.0 and 3.0–5.5 ppm were assigned to protons in flavonoid aglycone and 6- or 8-*C*-glycoside, respectively. Letter a labeled on peak indicates proton on C-6′. Figure S3: PLS score plot constructed from ^1^H-NMR spectral data of four different solvent extracts of OPL. The plot ellipse represents 95% hoteling T2 confidence. Figure S4: Permutation test results (100 permutations) for validation of PLS models built for (A) DPPH free radical scavenging, (B) NO free radical scavenging, (C) FRAP, (D) cell proliferation, and (E) cell migration activities.
