# Supplementary material for: Metabolite Characterization and Correlations with Antioxidant and Wound Healing Properties of Oil Palm (Elaeis guineensis Jacq.) Leaflets via 1H-NMR-Based Metabolomics Approach
Source: Molecules. 2020 Nov 30;25(23):5636. doi: 10.3390/molecules25235636 (PMC7731087; doi:10.3390/molecules25235636)
Supplement: Supplementary file 1 [file molecules-25-05636-s001.pdf]

molecules

MDPI

Supplementary Materials

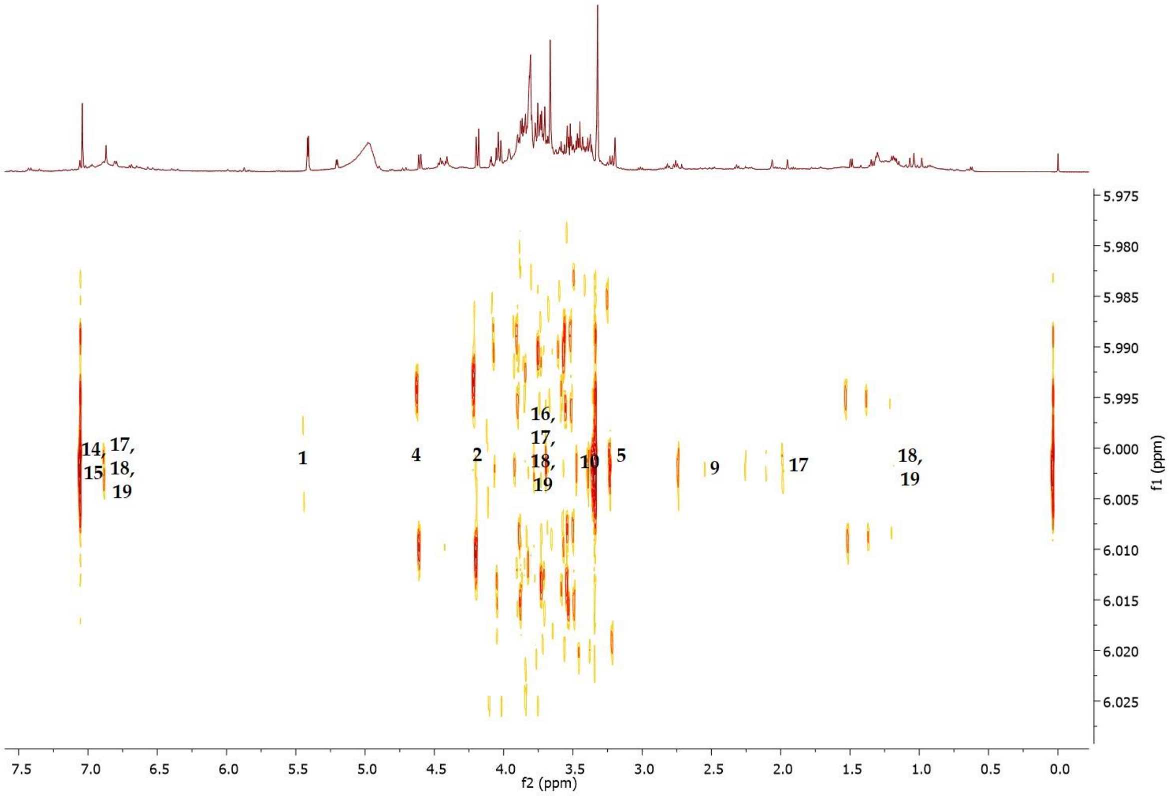

Figure S1 2D NMR ^{1}H (J-resolved) spectrum of methanolic extract of oil palm (*Elaeis guineensis* Jacq.) leaflet

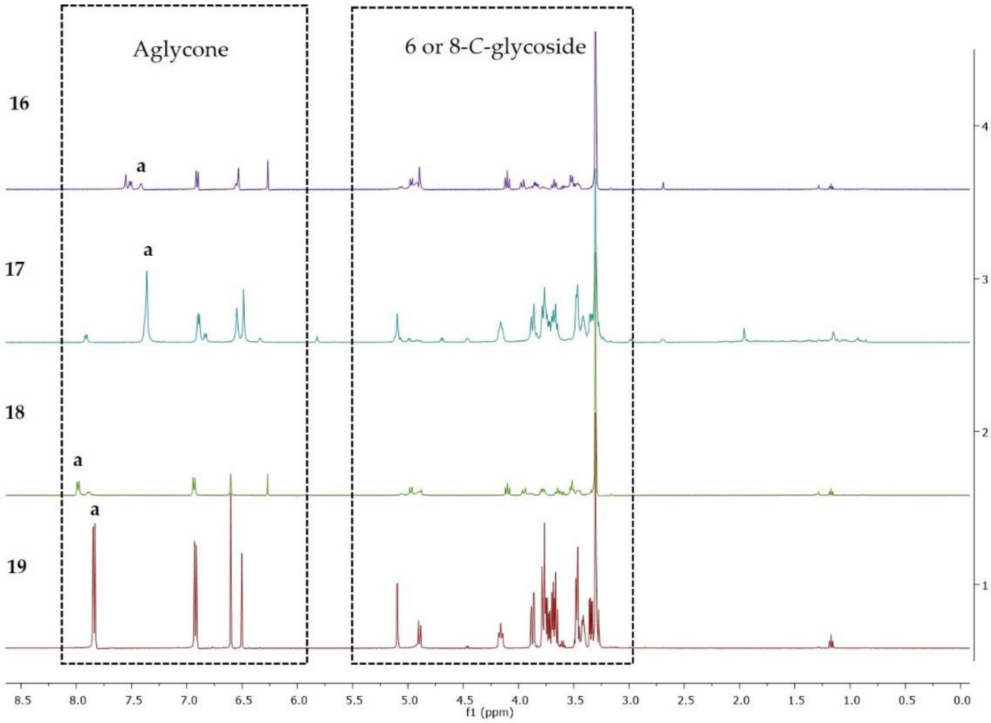

Figure S2 ^{1}H NMR spectra of commercial standards of orientin (**16**), isoorientin (**17**), vitexin (**18**) and isovitexin (**19**). Signals appeared in the region between 6.0-8.0 and 3.0-5.5 ppm were assigned to protons in flavonoid aglycone and 6- or 8-C-glycoside, respectively. Letter **a** labeled on peak indicates proton on C-6′.

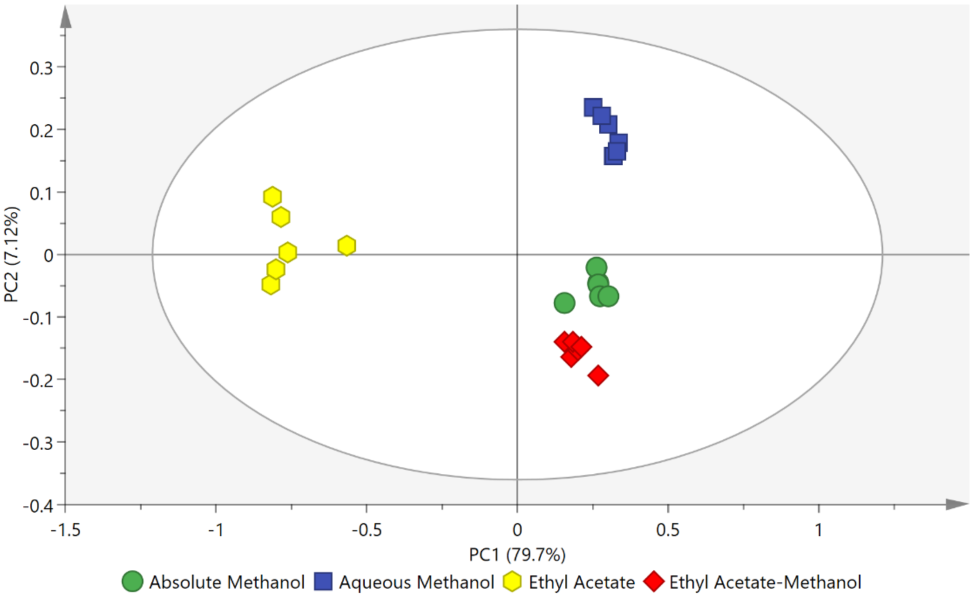

Figure S3 PLS score plot constructed from ^{1}H NMR spectral data of four different solvent extracts of OPL. The plot ellipse represents 95% hoteling T2 confidence.

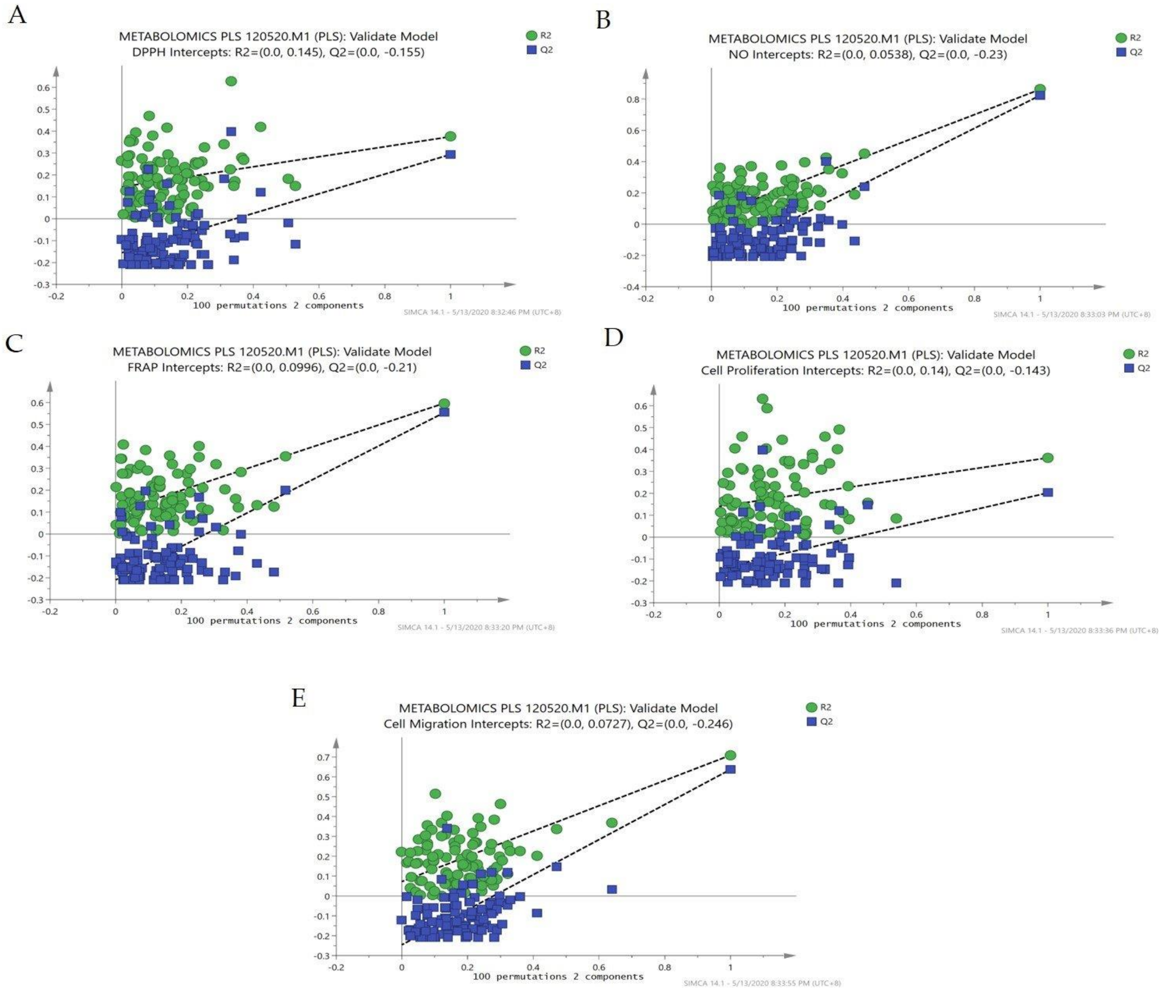

Figure S4 Permutation test results (100 permutations) for validation of PLS models built for (A) DPPH free radical scavenging, (B) NO free radical scavenging, (C) FRAP, (D) cell proliferation, and (E) cell migration activities.
